# Ethnicity and the diagnosis gap in liver disease: a population-based study

**DOI:** 10.3399/bjgp14X682273

**Published:** 2014-10-27

**Authors:** William Alazawi, Rohini Mathur, Kushala Abeysekera, Sally Hull, Kambiz Boomla, John Robson, Graham R Foster

**Affiliations:** The Liver Unit, The Blizard Institute, Barts & The London School of Medicine, London, UK.; Centre for Primary Care and Public Health, The Blizard Institute, Barts & The London School of Medicine, London, UK.; The Liver Unit, The Blizard Institute, Barts & The London School of Medicine, London, UK.; Centre for Primary Care and Public Health, The Blizard Institute, Barts & The London School of Medicine, London, UK.; Centre for Primary Care and Public Health, The Blizard Institute, Barts & The London School of Medicine, London, UK.; Centre for Primary Care and Public Health, The Blizard Institute, Barts & The London School of Medicine, London, UK.; The Liver Unit, The Blizard Institute, Barts & The London School of Medicine, London, UK.

**Keywords:** ethnicity, liver, liver disease, NAFLD, population

## Abstract

**Background:**

Liver disease is a major cause of morbidity and mortality worldwide. Large numbers of liver function tests (LFTs) are performed in primary care, with abnormal liver biochemistry a common finding. Non-alcoholic fatty liver disease (NAFLD) is the most common cause of chronic liver injury. Metabolic syndrome, common in people from South Asia, is an important risk factor for NAFLD.

**Aim:**

It is hypothesised that a large gap exists between numbers of patients with abnormal LFTs and those with recorded liver diagnoses, and that NAFLD is more common among adults of South Asian ethnic groups.

**Design and setting:**

A cross-sectional study of 690 683 adults in coterminous general practices in a region with high ethnic diversity.

**Method:**

Data were extracted on LFTs, liver disease, and process of care measures from computerised primary care medical records.

**Results:**

LFTs were performed on 218 032 patients, of whom 31 627 had elevated serum transaminases. The prevalence of abnormal LFTs was highest among individuals of Bangladeshi ethnicity. Of the patients with abnormal LFTs, 88.4% did not have a coded liver diagnosis. NAFLD was the most frequently recorded liver disease and was most common among Bangladeshi patients. In a multivariate analysis, independent risk factors for NAFLD included Bangladeshi ethnicity, diabetes, raised BMI, hypertension, and hypercholesterolaemia.

**Conclusion:**

Abnormal LFTs are common in the population, but are underinvestigated and often remain undiagnosed. Bangladeshi ethnicity is an important independent risk factor for NAFLD.

## INTRODUCTION

Hospital admissions and deaths from liver disease are rising across the Western world.^1^,^2^ Most epidemiological studies focus on prevalence rates of individual diseases in selected hospital populations^3^ as much early stage liver disease in primary care settings is asymptomatic. Alternative strategies include studying community cohorts, such as those found to have liver-related abnormalities on blood testing, or studying diagnostic prevalence in computerised primary care records.^4^–^7^

Early identification of liver disease is important for timely intervention. Diagnoses are usually made after measurement of liver biochemical tests, chiefly alanine transaminase (ALT) and aspartate aminotransferase (AST). Increasing numbers of liver function tests (LFTs) are performed in primary care^5^,^8^,^9^ for a variety of indications.^10^,^11^ Despite high rates of testing, a large number of patients continue to present to secondary care in the later, decompensated, stages of disease, suggesting that there are important gaps between detecting abnormalities, making diagnoses, and implementing treatment.

Abnormal LFTs are not universal in chronic liver disease. Long periods in the natural history of conditions such as non-alcoholic fatty liver disease (NAFLD) or chronic hepatitis B virus (HBV) infection are characterised by transaminase values within the reference range, masking ongoing liver injury.

In the UK the most common cause of liver dysfunction is NAFLD, affecting 10–30% of the general population, rising to 80–90% in obese patients.^3^,^12^–^16^ NAFLD is closely linked with other components of the metabolic syndrome: obesity, type 2 diabetes, hypercholesterolaemia, and hypertension.^17^ The prevalence of NAFLD in different ethnic groups living in developed countries has received little attention. The prevalence of hepatic steatosis was most common in Hispanic patients in an American study,^13^ and NAFLD may be more aggressive in those of Latino origin.^18^ Little is known of prevalence in other populations, but given the high rates of metabolic syndrome in South Asian populations,^19^ the authors hypothesise that NAFLD is more prevalent in these ethnic groups.

A regional database was used to identify patients with abnormal LFTs to estimate the burden of undiagnosed liver disease in primary care. Rates of recorded NAFLD were studied in patients from different ethnic groups to determine whether South Asian ethnicities are at increased risk.

## METHOD

The study was conducted in three east London boroughs (Tower Hamlets, Newham, and City & Hackney), where more than 50% of the population are from ethnic minority groups. Data were anonymised and extracted from the electronic record for all patients aged ≥18 years registered with the 150 out of 154 GP practices that use the EMIS Web clinical system (total 817 700). Data were managed according to UK NHS information governance requirements.

**How this fits in**

Abnormal liver function tests (LFTs) are a common finding in primary care, and in this study it is shown that many patients with such abnormalities have no recorded liver diagnoses. Ethnic group and components of the metabolic syndrome are among key independent risk factors for abnormal LFTs, and a large number of these patients are likely to have undiagnosed non-alcoholic fatty liver disease (NAFLD). NAFLD is the most common liver diagnosis in the general population and being of Bangladeshi, but not any other South Asian ethnicity, is identified as a novel independent risk factor for this condition.

Demographic variables included age, sex, ethnic group, and social deprivation. Self-reported ethnic group was collapsed into categories: white, Bangladeshi, Indian, Pakistani, African, Caribbean, and other (including mixed) and not stated (ethnicity not determined because of non-response or coding error). Social deprivation was classified according to the Townsend score.

Liver diagnoses included NAFLD, alcoholic liver disease, hepatitis A, B, C, D, and E, autoimmune hepatitis, primary biliary cirrhosis, primary sclerosing cholangitis, haemochromatosis, Wilson’s disease, alpha-1 antitrypsin deficiency, portal vein thrombosis, Budd Chiari syndrome, glycogen storage disorder, cholestasis of pregnancy, liver disease in pregnancy, and HELLP syndrome. Glandular fever and pre-eclampsia were included as causes of abnormal transaminases.

Comorbid diabetes, hypertension, and cardiovascular disease were recorded. Clinical care measures included viral serology completion, tobacco and alcohol consumption, and latest recorded values for body mass index (BMI), serum ALT, AST, and lipids. Use of drugs known to commonly cause abnormal LFTs was recorded: amiodarone, azathioprine, carbamazepine, methotrexate, phenytoin, antituberculous medications (isoniazid, pyrazinamide, rifampicin, ethambutol), and statins.

BMI values were collapsed into four categories of underweight (<18.5 kg/m^2^), normal weight (18.5–24.9 kg/m^2^), overweight (25.0–29.9 kg/m^2^), and obese (≥30.0 kg/m^2^). For Bangladeshi, Indian, and Pakistani patients, the category cut-offs were set at underweight (<18.5 kg/m^2^), normal weight (18.5–22.9 kg/m^2^), overweight (23.0–27.4 kg/m^2^), and obese (≥27.5 kg/m^2^).^20^ Alcohol use was categorised into within or greater than recommended limits (males ≤21 units/week, females ≤14 units/week), and ‘not recorded’. Statistical analyses were conducted using Stata (version 12).

## RESULTS

### Abnormal liver function in primary care

The study population comprised 690 683 adults (Table 1). Ethnicity was recorded for 93.1% of the adult population and for subsequent analyses, the focus was on the six most populous ethnic groups: white, Bangladeshi, Pakistani, Indian, African, and Caribbean. BMI was recorded for 89.2% of all adults, and units of alcohol/week for 91.0%.

**Table 1.** Demographic and clinical characteristics of the study population

	**Bangladeshi**	**Indian**	**Pakistani**	**White**	**African**	**Caribbean**	**Total population**
*n*	93 315	53 342	31 794	278 944	47 152	23 388	690 683
Mean age, years (SD)	36.5 (14.0)	38.6 (14.8)	37.2 (14.1)	41 (16.3)	40.8 (13.6)	49.7 (17.9)	39.5 (15.4)
Mean Townsend deprivation score (SD)	6.3 (1.7)	4.7 (1.8)	4.8 (1.7)	5.4 (1.9)	5.9 (1.8)	5.6 (1.8)	5.5 (1.9)
Female, *n* (%)	42 221 (45.2)	23 340 (43.8)	12 353 (38.9)	146 113 (52.4)	24 835 (52.7)	13 227 (56.6)	340 455 (49.3)
Male, *n* (%)	51 094 (54.8)	30 002 (56.2)	19 441 (61.1)	132 831 (47.6)	22 317 (47.3)	10 161 (43.4)	350 228 (50.7)
Diabetes, *n* (%)	11 554 (12.4)	5028 (9.4)	2991 (9.4)	12 666 (4.5)	3575 (7.6)	3618 (15.5)	47 232 (6.8)
Hypertension, *n* (%)	9599 (10.3)	6055 (11.4)	2981 (9.4)	29 910 (10.7)	8078 (17.1)	6540 (28.0)	76 704 (11.1)
**BMI**							
Underweight, *n* (%)	1482 (1.6)	6225 (11.7)	445 (1.4)	3933 (1.4)	247 (0.5)	149 (0.6)	9458 (1.4)
Normal, *n* (%)	10 352 (11.1)	10 811 (20.3)	3121 (9.8)	74 890 (26.8)	6154 (13.1)	3344 (14.3)	133 287 (19.3)
Overweight, *n* (%)	21 098 (22.6)	10 269 (19.3)	5636 (17.7)	45 331 (16.3)	9404 (19.9)	5112 (21.9)	120 060 (17.4)
Obese, *n* (%)	17 929 (19.2)	25 139 (47.1)	7441 (23.4)	36 211 (13.0)	10 367 (22.0)	6361 (27.2)	106 800 (15.5)

BMI = body mass index. SD = standard deviation.

**Figure 1.**
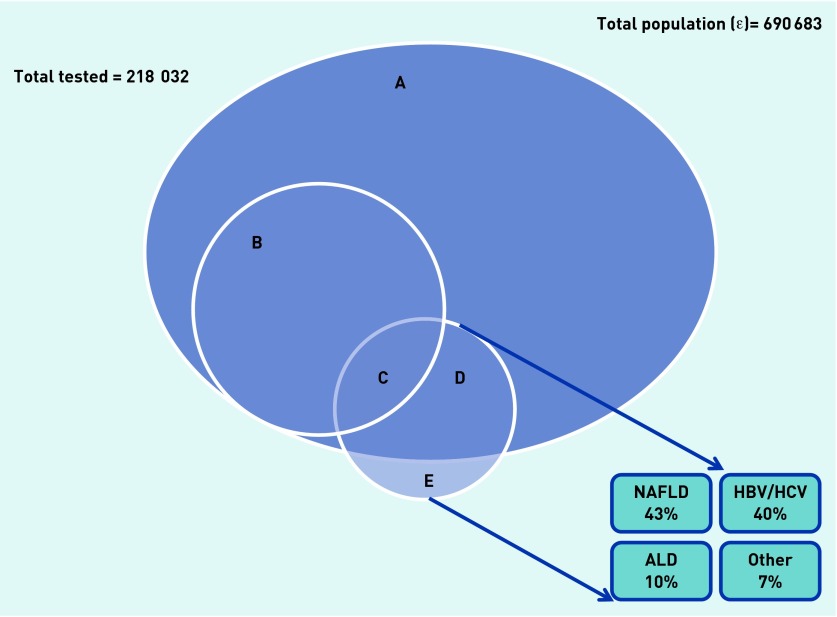
***Venn diagram showing patients with abnormal liver tests and most commonly recorded liver diagnoses. (A+B+C+D) = All adults tested (n = 218 032); Set A = Tested and normal LFTs (n = 196 360). (B+C) = Tested and abnormal LFTs (n = 31 672); Set B = Tested, abnormal LFTs but no diagnosis (n = 27 985); Set C = Tested, abnormal LFTs and liver diagnosis recorded (n = 3687). (C+D+E) = Liver diagnosis recorded; Set D = Tested, normal LFTs and liver diagnosis recorded (n = 4384); Set E = Not tested, liver diagnosis recorded (n =3965). This group of sets is further broken down into the proportions of patients with the common liver diagnoses. ALD = alcoholic liver disease. HBV = hepatitis B virus. HCV = hepatitis C virus.***

LFTs were performed for 31.6% of this population in the previous 2 years (*n =* 218 032 patients; Figure 1). The tested population was older than the general adult population (49.1 versus 39.4 years). Testing varied by ethnicity: 46.5% of adults of Caribbean ethnicity had liver testing, compared with 40.6% of Bangladeshi, 37.8% of Pakistani, 36.3% of Indian, 36.0% of African, and 28.6% of white adults. Of the tested population, 19.1% had diabetes (compared with 6.8% of the total adult population, *P*<0.001).

Among the tested population, 14.5% had an ALT or AST elevated above the upper limit of the normal range on at least one occasion during the 2-year period. The key demographic and clinical data for the populations with normal and abnormal LFTs are shown in Table 2. Within the total tested population, the mean ages of patients with normal and abnormal LFTs were comparable and most patients with abnormal LFTs were male. The prevalence of abnormal LFTs was highest among Bangladeshi (18.4%) and Pakistani (17.6%) adults, and lowest among adults of Caribbean ethnicity (10.2%), while the prevalence rates among patients of Indian (14.8%), white (13.5%) and African (11.8%) ethnicities were comparable. The mean ALT in the group of patients with raised transaminases was 67i U/ml (SD = 62.2, median 52, interquartile range [IQR] 24) and the mean AST was 65 iU/ml (SD 72.5, median 46, IQR 24).

**Table 2.** Demographic and clinical characteristics of tested patients^a^ with normal and abnormal liver function tests (LFTs)

**Variable**	**Normal LFTs (*N* = 186 360) *n***	**Abnormal LFTs (*N* = 31 672) *n* (%)**
Female	108 540	9596 (8.1)
Male	77 820	22 076 (22.1)
Bangladeshi	30 954	6962 (18.4)
Indian	16 507	2876 (14.8)
Pakistani	9917	2111 (17.6)
White	69 028	10 791 (13.5)
African	14 975	2004 (11.8)
Caribbean	9772	1112 (10.2)
Mean age, years (SD)	49.6 (17.4)	47.0 (14.3)
Diabetes	33 673	22 804 (40.4)
Hypertension	52 480	9226 (29.1)
Cardiovascular disease	11 968	6248 (34.2)

a n = 218 032. SD = standard deviation.

The odds of a finding of abnormal LFTs in the tested population were calculated for a range of potential risk factors in a multivariate analysis (Table 3). The odds of having abnormal LFTs in the tested population were similar in the Bangladeshi and white ethnic groups, and significantly reduced in patients of Indian, Pakistani, African, or Caribbean ethnicity. Key independent risk factors for abnormal LFTs include male sex, alcohol consumption in excess of recommended limits, and components of the metabolic syndrome (diagnosed diabetes, hypertension, raised BMI, and hypercholesterolaemia). There was a gradient of risk of abnormal LFTs with increasing BMI.

**Table 3.** Multivariate regression examining the odds of having abnormal liver function tests in the previous 2 years^a^

**Explanatory variable**	**Odds ratio**	**95% CI**	***P*-value**
**Ethnicity**			
White (ref)	1	–	
Bangladeshi	1.09	1.03 to 1.14	0.001
Indian	0.84	0.78 to 0.91	<0.001
Pakistani	0.92	0.85 to 1.00	0.055
African	0.77	0.71 to 0.82	<0.001
Caribbean	0.82	0.75 to 0.89	<0.001

Age (continuous)	0.98	0.97 to 0.98	<0.001

**Sex**			
Female (ref)	1	–	
Male	2.94	2.80 to 3.10	<0.001

Diagnosed diabetes	1.58	1.51 to 1.65	<0.001

Diagnosed hypertension	1.16	1.11 to 1.21	<0.001

Diagnosed CVD	0.90	0.84 to 0.95	0.001

**BMI category**			
Normal (ref)	1	–	
Underweight	0.84	0.70 to 0.99	0.042
Overweight	1.63	1.53 to 1.74	<0.001
Obese	2.44	2.27 to 2.62	<0.001

**Alcohol consumption**			
Safe (ref)	1	–	
Unsafe	1.92	1.80 to 2.05	<0.001

a n *= 125 429 out of 218 032 cases with full data sets. Adjusted for Townsend deprivation score, locality and clustered by practice (139 clusters). CVD = cardiovascular disease. ref = reference category.*

### Most liver abnormalities are undiagnosed

Of the 31 672 patients with abnormal transaminases, only 3687 (11.6%) had a liver-related diagnosis in the clinical record, the most common of which were NAFLD, viral infection, and alcoholic liver disease (Appendix 1).

There is no Read Code for drug-induced liver injury, but 900 patients were identified who were taking medication associated with abnormal LFTs (amiodarone, azathioprine, carbamazepine, methotrexate, phenytoin, and antituberculous medications). In the adult population, 87 532 patients (12.7%) were prescribed a statin. Of 27 985 patients who did not have a diagnosis, 11 111 were taking a statin.

Only 494 patients with abnormal LFTs had a recorded diagnosis of alcoholic liver disease (ALD). Alcohol usage was recorded in 28 802 (91.0%) adults with abnormal LFTs, of whom 3866 (13.4%) were drinking in excess of recommended limits. Therefore, in 3372 patients without a recorded diagnosis the abnormal LFTs were at least associated with alcohol.

Of patients with abnormal LFTs, 6026 (19.0%) were recorded as drinking within recommended limits and had undergone testing for viral hepatitis and did not have a positive result. This group represents a population that may have high rates of NAFLD and/or other chronic liver diseases.

### Prevalence of recorded diagnosis of liver disease

It was reasoned that a large number of patients with liver disease may not have abnormal LFTs. Patients with recorded liver-related diagnoses in the whole adult population were investigated therefore, irrespective of biochemical testing.

**Figure 2.**
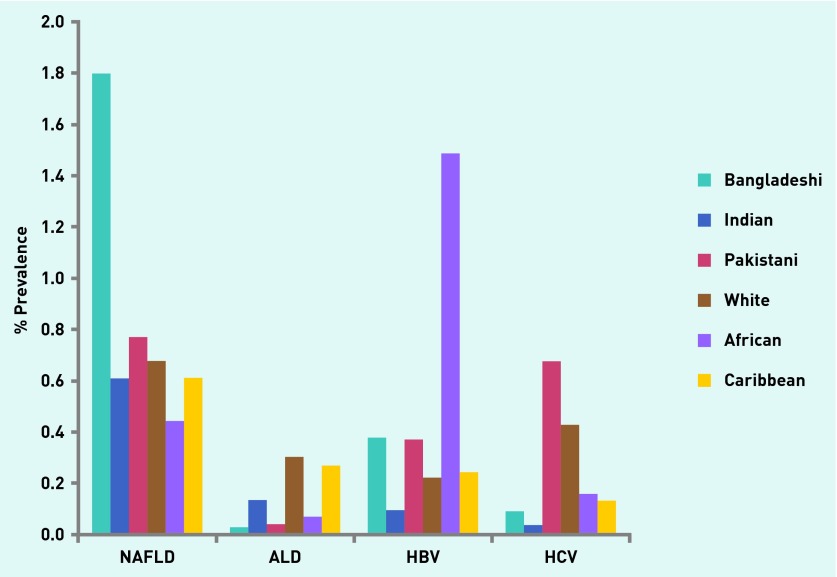
***Prevalence of NAFLD, ALD, HBV and HCV in different ethnic groups within the general population.***

A liver-related diagnosis was recorded in 12 239 (1.7%) of the total adult population. The most commonly recorded liver diagnosis was NAFLD in 42.9% (*n* = 5250), followed by HBV (23.8%, *n* = 2910), hepatitis C virus (HCV) infection (15.7%, *n* = 1922), and ALD (9.9%, *n* = 1215). Only 436 (3.6%) had more than one liver diagnosis. A diagnosis of ALD was most prevalent among people of white (0.3%), HBV in people of African (1.49%) and HCV in people of Pakistani (0.68%) ethnicity (Figure 2).

Not all patients with liver-related diagnoses had abnormal LFTs in the 2-year period of the study. Liver function tests had been performed in 82.5% of patients with NAFLD, of whom 55.7% had normal results.

### Risk factors for NAFLD

As NAFLD was the most prevalent liver disease, the risk factors for NAFLD in the general population were determined. Diagnosed NAFLD was significantly more prevalent among people of Bangladeshi ethnicity (1.8% of the adult population) than any other ethnic group, including other South Asian groups (Appendices 2 and 3). As expected, the prevalence of NAFLD was significantly lower in the African and Caribbean ethnic groups.

The odds of a diagnosis of NAFLD in the general adult population were calculated, by ethnicity, adjusting for a range of previously established risk factors (Table 4). The highest risk for NAFLD was in patients with raised BMI (with a gradient of risk of NAFLD with increasing BMI) and in patients with comorbid diabetes or hypertension. Other risk factors include hypertension but not sex, smoking, age, social deprivation, or a diagnosis of cardiovascular disease. Bangladeshi, but not Indian or Pakistani, ethnicity remained an independent risk factor for NAFLD in the adjusted analysis.

**Table 4.** Multivariate regression examining the odds of having diagnosed NAFLD^a^

**Explanatory variable**	**Odds ratio**	**95% CI**	***P*-value**
**Ethnicity**			
White (ref)	1		
Bangladeshi	1.86	1.56 to 2.23	<0.001
Indian	0.94	0.71 to 1.24	0.657
Pakistani	1.31	0.99 to 1.74	0.062
African	0.58	0.47 to 0.72	<0.001
Caribbean	0.45	0.35 to 0.58	<0.001

Age (continuous)	1.01	1.01 to 1.02	<0.001

**Sex**			
Female (ref)	1		
Male	1.02	0.95 to 1.09	0.574

Diagnosed diabetes	2.74	2.51 to 2.99	<0.001

Diagnosed hypertension	1.34	1.15 to 1.57	<0.001

Diagnosed CVD	0.84	0.75 to 0.95	0.007

**BMI category**			
Normal (ref)	1		
Underweight	0.55	0.25 to 1.23	0.148
Overweight	4.52	3.72 to 5.51	<0.001
Obese	9.59	7.77 to 11.8	<0.001

a n = 268 657 out of 690 683 cases with full data sets. Adjusted for Townsend deprivation score, alcohol consumption, locality and clustered by practice (139 clusters). CVD = cardiovascular disease. ref = reference category.

## DISCUSSION

### Summary

Abnormal liver tests are a common finding in primary care,^21^ yet this study shows that most patients with abnormal LFTs who are likely to have significant liver disease are undiagnosed.

Abnormal LFTs exist in 14.5% of the tested population and the marked male predilection may be related to alcohol consumption^22^ and HCV infection.^23^ Ethnicity is an independent risk factor for abnormal LFTs, with Bangladeshi and white patients at higher risk than other groups. Risk factors identified in the current study include raised BMI and a recorded diagnosis of diabetes, both of which are strongly associated with NAFLD.

More than half of patients with abnormal LFTs had no recorded aetiology or evidence of serological investigation, despite the fact that detection of even mild derangements in LFTs is a significant risk factor for liver disease, all-cause and liver-related mortality, and despite the higher risk of viral liver disease in a multiethnic population.^6^ Excess alcohol consumption was common among patients with abnormal LFTs (13.4%), which is lower than rates reported in other series, although it is important to remember that a significant proportion of patients in the present cohort were from religious and social backgrounds in which alcohol is rarely consumed or where consumption is stigmatised.

A large proportion of patients with recorded liver diagnoses did not have abnormal LFTs, and this highlights the limitation of transaminase testing as a screening tool for liver disease.^24^ Even patients with ALT 20–40 iU/ml are at increased risk of liver-related mortality and this is independent of the liver diagnosis.^25^ Furthermore, other key abnormalities in liver biochemistry were not recorded — gamma-glutamyltransferase or alkaline phosphatase — which may be elevated in the context of normal transaminases. Therefore, it is likely that although this study has identified patients with diagnosed liver disease and a probable cohort with undiagnosed significant liver disease, these data still underestimate the true burden of liver disease in the population.

### Strengths and limitations

This study is the largest community-based study of the association of ethnicity with LFTs and NAFLD to date, and was conducted in one of the most ethnically diverse regions of the UK. It benefits from high levels of data completeness for key characteristics: ethnicity, BMI, and alcohol consumption. Unlike other cohorts, the present data are derived from contiguous (unselected) GP practices across the region, and therefore include all adult patients. This includes practices that specialise in the care of homeless persons and injecting drug populations. Despite this, it is possible that data are lacking from unregistered patients who may be among the most at-risk sectors of society.

Although ALT was shown in the BALLETS study to be the best liver biochemistry test for the exclusion of significant hepatocellular disease,^26^ by focusing on transaminases and not alkaline phosphatases, patients with biliary disease or hepatobiliary tumours may have been overlooked, and therefore the true burden of liver disease in primary care may well be even higher than suggested here.

The EMIS database is principally a clinical tool and reflects current practice in primary care. There may be bias in data entry, therefore, towards mandatory or positive data. A priori clinical suspicion usually exists, which is not captured in the electronic record, before LFTs are requested. The present estimates are comparable with published data regarding the distribution of liver disease by ethnicity;^2^,^27^ HBV is most common among Africans, HCV among Pakistanis, and ALD among white people. However given the ethnic mix in the study population, the relatively low rates of viral testing in patients with abnormal LFTs but without a liver diagnosis suggest a diagnostic gap in the estimates of HBV and HCV infection rates.

It is not possible to evaluate the accuracy of a coded diagnosis of NAFLD, although it is likely that most were made after attendance at specialist liver clinics. Similarly, among the undiagnosed patients with abnormal LFTs, it was not possible to determine the reasons for the absence of a diagnostic code. In some, the abnormality may have resolved after a period of watchful waiting, or resolution of a concurrent illness.

### Comparison with existing literature

This study found that NAFLD was the most commonly recorded cause of abnormal LFTs in the population in keeping with data from others,^28^ although the prevalence rate in the present study is lower than estimates from series where the diagnosis was made radiologically in the general population or histologically in selected series of patients. This is partially explained by the high proportion of patients in the present cohort in whom NAFLD is the probable cause of abnormal LFTs but who do not have a liver diagnosis. It is also likely that a large proportion of patients with NAFLD have simply not been diagnosed yet.

The prevalence of recorded NAFLD varied considerably by ethnic group. To the authors' knowledge, this is the first study to identify Bangladeshi ethnicity as an independent risk factor for NAFLD, albeit in the context of low overall rates of liver diagnoses. Among Bangladeshis, there are high rates of type 2 diabetes and cardiovascular disease that may have a genetic basis. Increased prevalence may be related to intergenerational influences, early years and immigration impacts on lifestyle and health beliefs.^29^,^30^

### Implications for research and practice

Further work is required to understand the effect of ethnicity on the natural history of NAFLD and why Bangladeshis are at increased risk, and, in particular, the relative contributions of genetics, diet, social deprivation, and cultural health behaviours.

The high proportion of patients with abnormal LFTs without a diagnosis is a challenge to primary care clinicians. Among this group many will have a liver disease that is amenable to further management, which may prevent complications. Where a patient has had abnormal LFTs, normalisation does not necessarily mean that liver injury was transient, as shown by the high proportion of patients with NAFLD and normal LFTs in the previous 2 years.

The authors recommend that when abnormal liver tests are identified, every reasonable effort should be made to make a diagnosis and to record this. There is a need to evaluate the cost-effectiveness of increased investigations in primary care and onward referral to liver specialists, which may be in conflict with current financial pressures to reduce laboratory tests and traditional outpatient attendance. Therefore, the authors support the development of evidence-based guidelines for the investigation, referral, and management of patients with abnormal LFTs in the community, to ensure early identification of treatable disease.

## Figures and Tables

**Appendix 3.** ***Prevalence of NAFLD in patients in different age ranges by ethnic group.***

## Appendix

**Appendix 1.** Recorded liver diagnoses in patients with abnormal liver function tests

**Diagnosis**	***n***	**% of abnormal LFT population (N = 31 672)**
NAFLD	1918	6.06
Alcoholic liver disease	494	1.56
Hepatitis B	388	1.23
Hepatitis C	523	1.65
Pregnancy-related	210	0.66
Acute viral infection	178	0.57
Glandular fever	154	0.49
Autoimmune hepatitis	59	0.19
Primary biliary cirrhosis	41	0.13
Primary sclerosing cholangitis	20	0.06
Metabolic	58	0.18
Venous thrombosis	13	0.04

LFT = liver function tests. NAFLD = non-alcoholic fatty liver disease.

## Appendix

**Appendix 2.** Demographic and clinical characteristics of patients with a recorded diagnosis of NAFLD by ethnicity

	**Bangladeshi**	**Indian**	**Pakistani**	**White**	**African**	**Caribbean**	**Total**
*n*	1678	325	245	1889	209	143	5250
Prevalence, %	1.8	0.6	0.8	0.7	0.4	0.6	0.8
Mean age, years (SD)	36.5 (14.0)	38.6 (14.8)	37.2 (14.1)	41 (16.3)	40.8 (13.6)	49.7 (17.9)	39.5 (15.4)
Mean Townsend deprivation score (SD)	6.3 (1.7)	4.7 (1.8)	4.8 (1.7)	5.4 (1.9)	5.9 (1.8)	5.6 (1.8)	5.5 (1.9)
Diabetes, *n* (%)	827 (49.3)	125 (38.5)	85 (34.7)	587 (31.1)	76 (36.4)	68 (47.6)	1984 (37.8)
Hypertension, *n* (%)	558 (33.3)	108 (33.2)	79 (32.2)	815 (43.1)	94 (45.0)	90 (62.9)	2015 (38.4)
**BMI**							
Underweight, *n* (%)	0 (0.0)	2 (0.6)	0 (0.0)	5 (0.3)	0 (0.0)	0 (0.0)	9 (0.2)
Normal, *n* (%)	57 (3.4)	9 (2.8)	4 (1.6)	86 (4.6)	9 (4.3)	8 (5.6)	235 (4.5)
Overweight, *n* (%)	492 (29.3)	66 (20.3)	42 (17.1)	403 (21.3)	48 (23.0)	26 (18.2)	1277 (24.3)
Obese, *n* (%)	851 (50.7)	182 (56.0)	159 (64.9)	1005 (53.2)	120 (57.4)	91 (36.6)	2721 (51.8)

BMI = body mass index. SD = standard deviation.
